# The Broken Promise of Institutional Psychiatry: Sexuality, Women and Mental Illness in 1950s Lebanon

**DOI:** 10.1007/s11013-022-09786-1

**Published:** 2022-05-12

**Authors:** Lamia Moghnieh

**Affiliations:** Department of English, Germanic and Romance Studies, University of Copenhagen, Copenhagen, Denmark

**Keywords:** Psychiatry, Patients, Sexuality, Archival ethnography, Lebanon

## Abstract

This article traces the case of Hala, a woman chronic patient of the Lebanon Hospital for Mental and Nervous Disorders (LHMND) in late 1950s Lebanon. Her story reveals a conglomeration of actors, expertise and technologies that regulated both her sexuality and mental illness, as she was moved, returned, then moved again, from the care of the family to the care of the psychiatric institution. By reconstructing an ethnographic case of the story of Hala, the article tackles an under-investigated area of research at the intersection of subjectivity, sexuality, psychiatry and family life. The case of Hala illustrates an on-going tension in defining and diagnosing mental illness for women between two forms of care: institutional psychiatry on one hand—promising a quick return of patients to society—and the family on the other, with its own understandings of what constitutes abnormality for women. Having lived at the hospital for more than twenty years, Hala’s voice and experience provide a powerful contribution to the ethnographic history of psychiatry in Lebanon. The article tackles questions on competing psychiatric and social authorities and the formation of psychiatric subjectivities. It also provides methodological and ethical reflections on the use of archives when conducting ethnographic research on psychiatry from the global peripheries. The case of Hala illustrates the patient’s own experience of LHMND’s policies of social rehabilitation in the late 1950s. It adds to a broader understanding of the processes that have led to the pathologizing of sexuality in under-studied societies such as Lebanon and the Middle East.

## Introduction

In the fall of 1957, thirty-six-year-old Hala arrived with her sister to the admission ward of the Lebanon Hospital for Mental and Nervous Disorders (LHMND)^1^ in Hazmieh, Lebanon. Hala was under the impression that they were paying a visit to their other sister who had recently given birth at a hospital. As they went inside, she became more suspicious of her sister’s intentions. At the admission ward, the sister explained to the psychiatric nurse that Hala had recently become the talk of their hometown in Syria. She recounted in detail Hala’s abnormal tendencies, most of which focused on her outspoken sexual behaviors. The nurse translated all that was said from spoken Arabic to English, one of the official languages of the hospital, writing the information into the patient record file.

Later that day, Hala was admitted into the hospital’s private and third class level^2^ usually reserved for patients from lower socioeconomic status—despite belonging to a well-off family. Assessing her mental state at the time of her admission, the psychiatric nurse wrote: “She looks anxious but with a stupid smile on her face. No memory defects. Hallucinations or delusions, no insight into her problem.” Hala then received two diagnoses for her condition: schizophrenia and nymphomania. Her treatment plan included the administration of drugs—such as Chlorpromazine—and sedatives, coupled with electroconvulsive therapy; typical treatments used for psychosis at the time (LHMND 1955–1958).

A few months into her institutionalisation, Hala managed to escape from LHMND, but was brought back again by one of her siblings. Shortly after her escape, she was officially discharged from the hospital with a note in her file that says she has not been cured, yet she is now cooperative. Less than a month into her discharge, Hala was admitted again by her brother-in-law. In 1960, she was classified by the hospital administration as a ‘a chronic schizophrenic’, placing her in permanent institutionalisation. She would remain at LHMND for about twenty years under the financial care of the Lebanese government, after which she disappears from the archives entirely.

During her hospital stay, Hala wrote at least two letters to her siblings that were never sent but placed at the end of her patient record file. These letters provide a unique insight into her long-term hospital experience. It offers a narrative outside of the clinical frame that presents an intricate description of the mechanisms through which some non-normative women became permanently abandoned in psychiatric institutions. From Hala’s constant negotiations with her siblings and her pleads to return back to society, what becomes clear is the authority of the family itself in diagnosing and defining what is normal with that of psychiatry. In these letters, sexuality and non-conforming gender behaviours, not schizophrenia, surface as the main pathology that Hala promises her siblings over and over again to control and manage.

In this article, I trace the story of Hala in LHMND through two readings: first through the clinical gaze of institutional psychiatry, as manifested by her patient record file; and second through her own narrative about her relationship with her family and hospital experience. These two accounts, one in relation to institutional psychiatry and the other in relation to family, illustrate the experiences of institutionalised women patients and the management of non-normative sexuality in the 1950s Lebanon. They also chart the processes through which the psychiatric promise of social integration for patients suffering from mental illness remains unfulfilled.

In doing so, this article contributes to literature on the historiography of psychiatry in postcolonial and peripheral settings (i.e. Abi-Rached 2020; Antić 2022; Keller 2007; Kilroy-Marac 2019; Sadowsky 1999) and the psychiatric management of sexuality (Lunbeck 1994). I take sexuality as a contested site between psychiatry and the family, where patients’ voices can also be heard. By reading Hala’s story in dialogue with (and sometimes against) the biotechnologies of institutional psychiatry, Hala returns to being a central agent in discussions around (her) illness, sexuality and care.

Research on colonial psychiatry has highlighted the expansive and experimental reach of technological practices of domination on the mind, behaviour and personality of the colonised other (Studer 2015; Keller 2007; Fassin 2000; Mahone and Vaughan 2007; Sadowsky 1999). Postcolonial psychiatry sought to rethink and realign many of its core assumptions, such as the role of culture, race and society in mental health development and therapy (Antić 2021). In Lebanon, institutional psychiatry was introduced at the turn of the twentieth century with the founding of the first asylum in the Levant, “the Lebanon Hospital for the Insane”. A missionary project conceived by protestant-turned Quaker Theophilus Waldmeier, the asylum functioned based on a conglomeration of “civilising, evangelising and humanitarian” interventions to mental illness, as psychiatry was to become a therapeutic approach with spiritual and modern scientific components (Abi-Rached 2020:5). The Lebanon Hospital for the Insane soon transformed into a modern hospital under the name of “the Lebanon Hospital for Mental and Nervous Disorders” (LHMND). Until its closing in 1983, LHMND received a variety of patients, mostly admitted by family members, and some by religious constituencies and the police. Patients came from Lebanon and neighbouring Arab countries such as Palestine, Jordan and Syria, and occasionally from the United States, Greece, UK, Malta, Turkey, Norway and Sweden, among others (LHMND 1956).^3^

Abi-Rached (2020) argues that, unlike the history of colonial and postcolonial psychiatry in other contexts, the therapeutic practices of institutional psychiatry in Lebanon were constituted by a medley of local and international actors with various competing interests in Lebanon and in the institutional making of psychiatry. This led LHMND to become a pioneering and international hospital for mental health reform, integration and treatment by the end of World War II. In this article, I explore this proliferating period in the history of institutional psychiatry in Lebanon. However, I locate the sources of psychiatry’s cultural authority not only in its institutional power but in the discipline’s generative conceptual apparatuses, and its authoritative reach in society. Following Omnia El Shakry’s seminal work on psychoanalysis in Egypt (2017), I take psychiatry in Lebanon as a modern project that was constitutive of various kinds of tensions, traditions and hybridizations. I do so by highlighting these frictions when it comes to the relationship between institutional psychiatry, family and women and by studying how psychiatry coincides with and sometimes differs from other moral, cultural and ethical forms of authority.

## Tracing the Story of Hala in the Psychiatric Archives: Methodological and Ethical Considerations

Writing about patients in psychiatric institutions requires a process that allows for re-imagining and re-assembling clinical archives. The archives thus become “a repository of traces, gaps and silences” that, when centered, unsettle and challenge linear and progressive history of psychiatry (Kilroy-Marac 2019:28) that has often overlooked or framed patients’ illness narratives (Kleinman 1989). Studying the traces, and sometimes the deafening silences, of patient voices in the archives is a slippery process of extracting marginal narratives. These narratives emphasize the complexity and ethics of interpretations and of accessing archives as truth-making documents (Steedman 2002; Bradley 1999).

In this section, I reflect on the methodological and ethical questions that I faced when withdrawing patients narratives from the archives of psychiatric institutions, beyond merely finding patient writings and records. As a medical anthropologist and a mental health practitioner, my interest in the archives of Lebanese psychiatry first came from a methodological necessity to deepen my ethnographic research on contemporary psychiatry and its relation to other socio-material discourses that manage the meanings and iterations of suffering and affliction in Lebanon. I saw the act of excavating the history of Lebanese psychiatry as a return to the past to better contextualize the contemporary present, and construct a thicker description of the cultural authority of psychiatry in Lebanon.^4^

This led me first to the archival records of the Lebanon Hospital for Mental and Nervous Disorders (LHMND), an institution that has deeply influenced relations of normality and pathology, definitions of madness and the governance of mental illness in Lebanon and neighbouring Arab countries (Arzoumanian 2017; Abi-Rached 2020). I came across the archives as I was starting my ethnographic PhD research on humanitarian psychiatry and trauma in Lebanon in 2012, but officially started my archival research by the end of 2016 while working as a part-time lecturer at the American University of Beirut (AUB). Housed at the AUB’s private library,^5^ the archives contain a manifold of documents on the institution’s daily life, conference proceedings, the history of psychiatric nursing, mental health organisation and services, patient files, hospital everyday life correspondences and annual reports,^6^ among others.

In my reading of these documents, I adopted a methodology of archival ethnography that privileges the lifeworlds, socio-political iterations and imaginaries of madness, illness and treatment. I was intrigued by the informal and communal ways of remembering the hospital as another kind of dynamic archiving of psychiatry and illness that evokes a less institutional and progressivist history. My engagement with the archives of the LHMND was then a constant exercise in this dual reading, or a reading of a duality between communal and institutional management of madness and illness.

Archival ethnography is formest a methodology that takes the archives as fieldwork, where social and cultural life, common sense and the mundane, and sociopolitical factors are emphasized and analysed (Decker and McKinlay 2020). In the case of institutional archives such as LHMND, ethnography can be a useful analytical tool (i.e. a way of listening, reading and engaging) that seeks to disrupt and critique the particular forms of knowledge and styles of reasoning that define a particular archive (Osborne 1999). Looking at the archives of LHMND as sites where madness is debated, contested, negotiated and expressed is an ethnographic question that attends to both communal and psychiatric practices of illness and healing. Archival ethnography also gives space for the researcher’s reflexivity and positionality to emerge as a critical form of knowledge-making about the multiplicity of voices and representations in institutional archives. Lastly, and as the final part of this section illustrates, ethnography is a form of critical writing that has the ability to provide an ethical form of narration about vulnerable groups such as institutionalised patients.

My engagement with the archives of psychiatric institutions is also strongly influenced by the recent “archival turn” in the Middle East amidst revolutions, protests civil unrest and war (Ghazaleh 2019; El Shakry 2015). The archives became a contested site for truth-making claims where nationalist and colonial histories can be told differently. Faced with the hegemonic, violent and inaccessible nation-state, and with the imperial and colonial looting of national archives, there has been a proliferation of alternative forms of archival practices by Middle Eastern scholars, activists and artists. The growing interest in accessing, studying and de-mystifying archival documents is inspired by many approaches such as feminist theory, history from below, oral history, interviews, fiction writing and family history.^7^ They produce narratives that counter mainstream accounts of national and colonial history. They also aim at recentering discursively invisible and silenced narratives back into history by re-enacting forgotten and hidden events, people and ideas into the present. The turn towards archives in the Middle East must also be about re-imagining the postcolonial archive itself as inclusive of multiple intellectual and political traditions and debates that have long been thought to be in opposition to one another (El Shakry 2015).

Archives and archiving as truth-making in the Middle East have raised important debates over the ‘proper’ authoritative way of accessing the archives, both physically and epistemologically. The roundtable discussion on “Archive as resistance” held at the Arab Council for the Social Sciences conference in 2019 in Beirut have revealed a lot of tension among scholars over the ability of anthropologists and feminist researchers—rather than historians and physicians—to read and decipher institutional and psychiatric archives. At the core of the questions raised in the roundtable were anxieties over authority: the authority of a discipline (only historians can unearth archives into a historical narrative; only medical doctors and psychiatrists can read and understand clinical archives), and the authority of the archives themselves, as factual documents, material fragments and a voicing of History, not as narratives that can be reassembled and reimagined as part of multiple forms of traditions, desires and projects.

In their own turn, archives of psychiatric institutions, if read only through their own authority, produce a constrained and restricted representation of patients along the line of psychiatric pathology and cure. Yet, patient records remain an important site to investigate the clinical interview as a site of knowledge-making and reform, where patients can return as actors and agents.

This article traces the stories of the patients *in relation* to institutional psychiatry and the family. It seeks to uncover the stories of those whose voices are constitutive of their own madness, voices that are constantly described in the archives as undecipherable, nonsensical, noisy, loud and confusing. Their stories are translated and made legible by nurses and psychiatrists only through their psychiatric diagnoses and their treatment prognoses, but also along descriptive terms like “restless, talkative, smiles without any reason, affect is blank, cries a lot, obedient and helpful”. These markers are often found in nurses and doctors’ notes and constitute an institutional view of the patient herself. In the patient records files, there are sometimes a sample of patients’ letters, their own writings and scribbles, some of them are directed to the hospital staff, others are addressed to family members or are personal reflections.

These writings, usually placed at the end of the file, serve as more evidentiary documents—alongside clinical and medical descriptions—to patients’ mental illness, and again, become constitutive of the psychiatric narrative of madness. The question of excavating patients’ voices from “total institutions” (Goffman 1961) therefore requires a different reading in/of the archives. It is first and foremost, an ability to listen to voices in the archives beyond the clinical frame. This is not a methodology about social construction of mental illness nor is it an invitation for doubting the diagnosis itself and assume that psychiatry is only about institutionalisation. Rather, it is an approach that departs from this binary that has long demarcated the social study of psychiatry. For example, my engagement with the archives is not to prove or disprove wether Hala actually had schizophrenia or whether she was institutionalised because of her sexuality, nor is it to simply add to the growing and important historical literature on patient narratives in/of psychiatric hospitals. I am more interested in reading institutional psychiatry’s practices of care and expertise from the point of view of her subjectivity, narrative and illness (Biehl 2005), tracing how the social, cultural and psychiatric management of ‘abnormal women’ and their mental illness came together to make Hala a chronic and long-term resident of LHMND, and what that did to her sense of self and voice.

### Ethics, Truth-Telling and the Ethnographic Case

In their article “Madness in the Archives: Anonymity, Ethics and Mental Health History Research” (2012), Wright and Saucier examine the ethical responsibilities and practices of historians studying mental health from the patient’s point of view. Amidst what they call a historiographical trend in telling patients’ stories, but also a general dearth in publishing about the ethics of this kind of research, they explore emergent tensions between uncovering forgotten voices from the archives and preserving the anonymity of institutionalised patients (2012).

Among the ethical tensions discussed, I have particularly struggled with the tension between the act of naming forgotten and silenced patients as a process of decolonisation that destigmatizes mental illness and the duty to anonymise and hide patients’ identities to preserve privacy and confidentiality (Wright and Saucier 2012). At the core of this tension is a debate on whether truth-telling practices can actually provide a form of liberation and justice. In thinking about the ethics in my own approach, I turn again to ethnography as a process of reflexive writing on my own position as researcher and mental health practitioner in the archives. My ethnographic reading of the archives taught me how to approach them less as sites of truth-telling where “uncovered” voices can provide historical accuracy and justice, than a place that holds a multiplicity of circulating and vibrant narratives.

In this sense, I tell the story of Hala as an ethnographic case (Yates-Doerr and Labuski 2017), or a composite account (Corman 2021**)** that, much like the rich history of “thinking in cases” (Forrester 1996), has the potentiality to instruct, evoke and situate the marginal, the details and the narration in relation to other forms of narratives (Yates-Doerr and Labuski 2017). Therefore, I am not interested in telling of the story of Hala as a historically accurate and factual story, nor do I feel that I have the authority to do so. Instead, I am engaged in writing the story of Hala as a lifeworld and affective narrative that speaks to / about women patients in Lebanon and their relation to institutional psychiatry. I do that first by using a pseudonym for Hala, and for other patients mentioned in the article, hiding and masking social demographics and providing general information about the dates of admission and hospital stay. I also do that by sometimes combining Hala’s story with the stories of other women patients from the same period. The narrative I construct of Hala is a critical story that provides insight over the relation between institutional psychiatry, women, and the family. This is how I try to walk the critical line between the “right to research” and “duty to protect” (Wright and Saucier 2012:67).

## Experiencing the Resocialization Project: New Policies in Everyday Hospital Life

At the time of Hala’s admission in 1957, LHMND was undergoing radical transformations in its ageing infrastructures and therapeutic policies (LHMND 1956, 1957). Rehabilitation of buildings, gardens, cafeteria, water and sewage systems were underway to reflect a new phase in the life of the institution. New wards and therapeutic units were added such as the Center for Occupational Therapy, the Social Center, and a permanent center for Electroencephalography (EEG). These developments all had a new aim: to prepare institutionalised patients to be integrated back into society. They specifically targeted the mental and physical health of the poorer and more chronic patients of the hospital, providing a way out of the hospital and back into society (LHMND 1956).

The problem and burden of “the chronic patient” have been a point of debate at LHMND as early as 1908, seven years after its opening. Now, with the development of new therapeutics and policies, the psychiatric promise of reintegration was stronger than ever. In the words of the hospital’s physician superintendent Dr. Manugian “in the near future the “chronic” wards will be a thing of the past in the hospital” (LHMND 1956:8).

One can read this new area in the history of institutional psychiatry in relation to a global and national interest in mental health services and care. The reorganisation of mental health after World War II by the World Health Organization (WHO), placed the Lebanese hospital as an actor in the global and Eastern Mediterranean management of mental health service and policy. On the national level, expansion in mental health practice was underway, with the establishment of new psychiatric hospitals such as the “Hospital Psychiatrique de la Croix”, commonly known as Deir-El-Saleeb, in 1951, and Dar El Azaja Al Islamiyya in 1961 (Katchadourian 1980; LHMND 1957). The involvement of the Lebanese state in institutional mental health care, especially the Ministry of Health and the Ministry of Social Affairs, became more tangible, providing donations and grants to psychiatric institutions (LHMND 1956) and increasing the rate of pay for public assistance from 425 Lebanese Pounds (around 48£) to 525 Lebanese Pounds (around 59.5£) per patient per day in 1960 (LHMND 1960). At LHMND, the change of name of the hospital from “Lebanon Hospital for mental disorders” to “Lebanon Hospital for Mental and Nervous disorders” in 1951 marked an institutional inclination towards new kinds of diagnostics and therapeutics**.** By the late 1950s, LHMND was clearly engaging in modernising the image of institutional psychiatry as more than just an asylum for the mentally ill, but as therapeutic intervention that promises a successful return to social life.

In many ways, Hala’s case and experience at LHMND embody this new psychiatric turn towards the social. The narrative about Hala, as described in the introduction, was part of her social history record in the patient file, a new diagnostic technology that facilitated institutional psychiatry’s reach into social and family life. It included Hala’s personal and family history, personality traits, social habits and previous personal and family medical history. These admission interviews were usually conducted by the psychiatric nurse. By 1956, LHMND had also employed its first psychiatric social worker whose job was to conduct interviews and visits with family members, keeping constant communication between hospital and family (LHMND 1956). Following the Social History Guide, a new document prepared by the hospital to guide nurses and social workers on gathering information on the social life of patients, Hala’s detailed life was recorded, especially her sexual behaviors and relationships with men.

Hala’s double diagnosis of nymphomania and schizophrenia also reflects this psychiatric interest in managing the social. In her book *The Psychiatric Persuasion: Knowledge, Gender, and Power in Modern America* (1994), Elizabeth Lunbeck describes nymphomania as the pathology of the over-sexualized woman and the ‘bad girl’ who oversteps and transgresses social and gender norms towards satisfying (but never able to do so) each and every sexual desire possible. Nymphomania and hysteria (the pathology of the ‘good girl’ who suppresses all sexual desire for the ability to comply with impossible gender and social expectations) both constituted the psychopathology of American women in the late nineteenth century and early twentieth century societies (Lunbeck 1994). It is, however, peculiar that the diagnosis of nymphomania was still used in the late 1950s at LHMND, as it does not seem to fit with the new forms of therapeutics nor with the diagnostics adopted by the hospital at the time.^8^ Nymphomania seems to have served more as a social diagnosis than a clinical one, used by the nurse to describe Hala’s social pathologies. Additionally, a longitudinal reading of Hala’s patient record shows that her outspoken and impulsive sexuality, first described as nymphomania in her record, was progressively translated and classified as a symptom of schizophrenia proper, and later on as an indication for chronic schizophrenia.

Yet, Hala’s sexuality and desires clearly remained to be one of the pathologies that were observed, recorded and treated at the hospital. During her long stay, nurses kept records of when Hala ‘chased after’, ‘continued to harrass’ and speak with men patients, the amount of time she cursed during the week, and her impulsive behaviours. This psychiatric management of women’s sexuality was not unique to Hala’s case. By the time Hala was admitted, sexuality represented one of the domains for psychiatric scrutiny at LHMND, joining other forms of everyday and social distresses like marriage, specific kinds of manhood, crime, war, and drug use. Other cases of at least two women patients can be detected in the archive, where sexuality and socially inappropriate sexual conducts played a role in their diagnoses and treatments. The case of Joseline for example echoes Hala’s diagnosis, in that she was admitted in the 1960s for delusions and behavioural disorders such as returning late at night without telling her parents where she was, and having men wait for her outside her parents’ house.

### Resocializing Sexuality, Managing Schizophrenia

As an incoming patient, Hala was the beneficiary of three intersecting new policies at LHMND: (1) a commitment to a chemical-social treatment, (2) an “open door” policy and (3) the Resocialization Project (LHMND 1956, 1957, 1958). These policies were translated in every aspect of the hospital from building, bedding, clothing, windows and diet (LHMND 1956). In terms of therapeutic management, LHMND had been experimenting with stabilisers like Chlorpromazine with psychotic patients, and for the general management of psychiatric disturbance (Majerus 2016; Ban 2007). Hala’s treatment coupled Chlorpromazine and Electroconvulsive therapy (ECT) with social therapy in the form of occupational therapy, habit training and Ergotherapy. Her weekly schedule consisted of engaging in several occupational and recreational activities, socialisation with other patients and visitors at the newly formed Social Centre, and going on outdoor trips and picnics in various sites in Lebanon. In occupational therapy, she was learning different kinds of laboured activities such as baking and sowing, while other patients engaged in carpentry, and helped with renovations in the gardens. At the centre, Hala would participate in a weekly program that included movie nights and social parties. These activities were gender specific in that they mimicked the social fabric from which these patients came.

The combined chemical-social treatment also worked to facilitate a second, policy, “the open door policy” that allowed patients to have more freedom of movement in and out of the hospital. Stabilisers helped suppress the excitement of patients at this new-found freedom while they became more familiar with receiving visitors and becoming exposed to social life. For Hala, this meant open spaces for socialisation, better bedding and clothes, a more nutritious and improved diet, a brighter room, the removal of irons from windows and doors and the possibility of escaping from the hospital, which she did shortly after her admission, only to be brought back again by her siblings.

Both the chemical-social treatment and the “open door” policy were part of the Resocialization Project at the hospital that focused on educating family and patients on the importance of reintegration into society, all the while increasing the number of discharged cases. This was done with a new kind of patient management that associated the release of patients not with a cure, but with the extent in which they were “socially recovered” (not cured but socially adaptive), “relieved” (where some symptoms have being relieved), or “not improved” (no significant changes). Shortly after her failed escape, Hala was classified as a “socially recovered” patient and was discharged to her family with the idea that she can continue her recovery at home, while still being talkative, having an incoherent speech and an infantile behaviour.

## “I Strongly Demand My Freedom”: Kinship, Subjectivity and Becoming a Chronic Patient of Psychiatry

In the late 1960s, the associate regional health director for mental health at the National Institute of Mental Health (NIMH), John Elderkin Bell, conducted a field visit to LHMND as part of his international research on the role of the family during the hospitalisation of a relative with mental illness.^9^ In his field visit, Bell described the hospital as beautiful and skilfully landscaped, buzzing with visitors—both family members and strangers coming from the neighbourhood or the city, Beirut—who were enjoying the hospital gardens, equipped with tables and benches and toilets (Bell et al. 1969). Bell enthusiastically observed the regular family visitation days held at the hospital on Wednesdays and Sundays, where the staff can attend to families, answer their questions and keep them updated about the progress of the patient. Some of the patients also had family members live with them for a while during their hospitalisation. A decade after the implementation of the Resocialization Project and open door policy, Bell found the hospital engaging in an “aggressive program to encourage family participation” (Bell et al. 1969:81) and promoting a form of rehabilitation that extended to the hospital’s outdoor grounds and gardens. This was done to lessen the isolation of the institution and its patients, promote the continuity of family relationship, destigmatize mental illness and reintegrate patients back into society.

Bell notes, however, that around one third to one half of the patients did not receive any visitors during their hospitalisation, especially the “chronically ill women patients”, who seem to have been the most abandoned. Hala belonged to this group of institutionalised women. Despite the reintegration policies and treatments, Hala lasted two months at home before she was re-admitted again. Her sexuality, not schizophrenia, remained the main complaint of her family as they noted that she resumed her previous activities such as going out to meet men and being publicly denounced in their hometown**.** In her record file, nurses noted the many times Hala was found crying because ‘she wants to go home’ and because ‘no one comes to visit her’. Diagnosed with ‘chronic schizophrenia’ in 1960 after her failed discharge as a socially recovered patient, Hala’s chances to reintegrate back into society became less of a possibility. She continued nonetheless to participate in the social integration activities such as baking and going to the hospital’s Social Centre; a centre built in 1957 particularly for those whose families do not visit.

A reading of the two letters that Hala wrote to her siblings provides an understanding of how she experienced the therapies at the hospital, what she imagined her return to society would be like and her devastating transformation from an acute patient, where the release from the hospital was possible, to a chronic patient of LHMND. Both letters were never received by family members, either because of a change in address, distance or other reasons. Placed in her file, the letters were incorporated as further evidence of Hala’s mental illness. Both letters are not dated, yet when compared together, it is clear that the first letter was written early on after her hospitalisation, while in the second letter, Hala mentions that around twenty years have passed since she has seen her family.

Hala addressed the first letter to her younger brothers. In a very coherent, agentive and strategic voice, she starts the letter with loving words for her brothers, reminding them of their kinship bonds and the good times that they have spent together. As Hala tries to negotiate her release from the hospital, one could not help but listen to her strong, poised and agentive voice, demanding her freedom (“I strongly demand my freedom”), insisting that she is not crazy and pleading with her brothers alongside normative gender roles.

In the letter, Hala negotiated her release by suggesting numerous ways in which she could return to society and assume socially acceptable and respectable gender roles: she could become a maid in her brothers’ houses, serving them from day to night, she could learn how to use the typewriter and join the women’s workforce in Syria, she could also become a housewife and a mother and run her own household. Hala also reminds her brothers of their prestigious and high social class and education level, to which she too belongs. How could they, then, sleep peacefully at night, when they have locked her in with *“al majanin*” (the crazy people), knowing very well that she was neither sick nor crazy. “I know the reason why I am in this hospital”, Hala wrote, “it is because you want to take away my inheritance: you have assumed my death and have inherited me while I am still alive”, she added.

Written at least twenty years after her admission the second letter, is addressed to her sister. The letter shows a devastating transformation in Hala’s subjectivity and voice. Now a chronic patient, Hala’s words are many times incoherent, and her sense of self appears as shattered. The difference in her voice between the two letters is indeed striking and is a commentary on the lived experience of chronic institutionalization and its impact on subjectivity.

Hala showers her sister with loving words and prayers. You have changed, she writes, ever since you gave birth to your last child, your voice and speech are not the same anymore. Perhaps *you* have “*marad Niswan”* (women’s illness), perhaps *you* need to come to the hospital too, there are many good doctors who will give you medications and needles for one or two weeks and then will cure and discharge you, and then maybe you, your husband and children can take me back with you.

Projecting her own story onto her sister, Hala ironically describes the Resocialization Project as a simple course of treatment than ends with women returning back to society. In the letter, she also writes, rather incoherently, about the number of cakes she baked and clothes she sowed in Occupational Therapy over the years, naming the doctors and nurses who ate and took them. Accumulating cream cakes, chocolate cakes, sweaters and dresses, all became a form of isolated and almost wasteful labor, absurdly dispersed onto doctors and nurses every week while Hala remained at the hospital.

Still, Hala tries again to negotiate her release to her sister by suggesting a useful, productive and gender appropriate role for her in society, such as working at her sister’s house and taking care of the children. Having lived at the hospital for twenty years, Hala had now acquired a deep knowledge of the ins and outs of the hospital and of which patient will be able to leave and which will become a chronic patient. She bluntly writes that “to be imprisoned [at the hospital] entirely depends on the will of the family. Whoever wants to release [patients] from the hospital they [the doctors] will quickly release them and if their family wants to leave them at the hospital then they will be left there.”

As Hala has clearly experienced, it is ultimately the family who decides when one can leave the hospital. Referring specifically here to “the chronic cases” of the hospital, those who have already spent so much time institutionalized that they became the hospital’s own, Hala insists in her letter that convincing the family, not the psychiatrist nor the occupational therapist, that she could be a productive labor force, a proper housewife or a maid for her brothers and sisters, was the only way for her to be released from the hospital. The issue of inheritance that she mentions is also telling of the materiality of the social and legal death of permanent women residents of psychiatric hospitals.

## Patients of Institutional Psychiatry: Rethinking the Relationship between Women and Institutional Psychiatry

The story of Hala at LHMND is telling of the faltering relationship between institutional psychiatry, women and mental illness in mid-twentieth century Lebanon. Her case reveals a conglomeration of actors, expertise and technologies that regulated both her sexuality and mental illness, as she was moved, returned then removed again, from the care of the family, to the care of the psychiatric hospital. In this article, I presented two readings of Hala’s story, one in relation to institutional psychiatry, and one in relation to her family.

Having lived at the hospital for more than twenty years, Hala’s voice and experience provide a powerful contribution to the ethnographic history of psychiatry in Lebanon. By reconstructing an ethnographic case of the story of Hala, the article tackles an under-investigated area of research at the intersection of subjectivity, sexuality, psychiatry and family life. It also provides methodological and ethical reflections on the use of archives when conducting ethnographic research on psychiatry from the global peripheries. Placing the institutional narrative of LHMND in dialogue with Hala's own narrative and kin relations, the article tackles questions on competing psychiatric and social authorities and the formation of psychiatric subjectivities. It contributes to a broader understanding of the processes that have led to the pathologization of sexuality in under-studied societies like Lebanon.

More specifically, the case of Hala provides three contributions. First, it enacts in praxis the lived experiences of the hospital’s reform policies. In many ways, the case of Hala at LHMND provides an interesting insight into the practices through which new hospital policies in the late 1950s Lebanon, and the promise of social reintegration, were implemented, and how, despite these initiatives, Hala became a chronic patient. Second, the case centres the family as the main diagnostician of what constitutes mental illness, where sexuality became a contested site that determined the kind of therapeutic subject allowed to return back to society. It exposes how Hala was governed by both psychiatry and the family, sometimes in contradictory and opposing ways. As Hala was being treated for both her sexuality and mental illness, there was a shared (yet different) interest in managing and treating her. For her siblings, Hala’s sexuality was the main abnormality that required treatment at the hospital. For the hospital, mental illness became entangled with Hala’s sexuality as her social and sexual life were reduced to symptoms used as further evidence for her schizophrenic condition.

This, I argue, is an interesting dynamic, as LHMND was the first institution in Lebanon to allow a distinctive shift in the governance of mental illness from the care of the family to the care of the psychiatric institution (Abi-Rached 2020). The case of Hala demonstrates on-going tensions and negotiations of definitions and diagnoses of mental illness for women between these two forms of care: a psychiatric promise of reform and rehabilitation on one hand and the family on the other, with its own classifications and understandings of what constitutes mental illness for women. The problem of the permanent psychiatric patient, an issue that preoccupied institutional psychiatry on a global scale, appears here as a product of this tension.

As Michel Foucault’s work on subjectification has shown, European modernity was predicated on a reconfiguration of power at the level of all institutions leading to the creation of the modern subject (Foucault 1990). The story of Hala demonstrates how psychiatry did not always imagine or create the same normalized subject as that of the family, especially when it came to women and their sexualities. This is not to argue that the case of institutional psychiatry in Lebanon produced a non-modern or pre-modern form of governance. Nor do I argue that this tension between family and psychiatry is one of contestation of western psychiatry as a colonial or foreign form of knowledge and practice. Instead, the case of Hala invites more attention to the process of making a chronic patient, that includes various tensions, frictions and debates between different forms of subjectification by institutional psychiatry and the family.

Finally, many aspects of Hala’s story echo the narratives of other women admitted to psychiatric institutions in different time periods in Lebanon, highlighting the need to further explore the relationship between institutional psychiatry, women, and mental illness. One narrative is that of the famous case of May Ziadeh—the Nahda intellectual, and literary writer —who was admitted to the same hospital in 1936 by her cousin because of inheritance issues (Kuzbari 1987). The second is also a public figure: Darina Al Joundi, Lebanese actress and writer, who wrote about how she was forcefully admitted in 1996 to Dair Al Salib hospital by her brother-in-law because of a loose sexuality and religious blasphemy during her father’s funeral (Joundi 2013). Hala’s story echoes in both these stories, inviting our attention to the processes through which certain women are institutionalised in psychiatric hospitals. Unlike Hala however, both May and Darina managed to be discharged from the hospital. May Ziade had to do a public lecture at the American University of Beirut (AUB), demonstrating her intellectual ability and rational thinking to psychiatrists and lawyers who attended the lecture. Darina el Joundi was also discharged by her family shortly after her hospital stay. As Hala repeatedly and poignantly remarked, one must account for the implication of kinship, the family and the social in the ways in which mental illness is classified and cured.

## Footnotes

1 The hospital was popularly known as Asfouriyeh, based on the name of the land where it was established.

2 At the time of her admission, the Beirut Executive Committee of the hospital had decided to increase private patients’ fees in all class levels. Class I fees from 450 LP (Lebanese Pound) per month (51£) to 500 LP per month (£ 57), Class II from 275 LP (31£) to 300 LP (£ 34) and class III from 150 LP (17£) to 175 LP (£ 20) (LHMND 1959).

3 After World War II, the institution witnessed an increase in patients’ admissions, Syrians being the second most admitted patients after the local Lebanese (see LHMND 1950, 1951, 1952, 1953, 1954, 1955, 1956, 1957, 1958, 1959, 1960).

4 This, of course is not new. Anthropologists have continuously turned towards the archives to study and think about hegemony, bureaucracy and empire (see for example Povinelli 2011 and Zeitlyn 2012), and to enrich their ethnographies.

5 Another part of the LHMND’s archive is hosted at SOAS’s library.

6 The hospital’s annual reports have been digitalised and made public by the AUB library https://lib-webarchive.aub.edu.lb/BorreLudvigsen/https://almashriq.hiof.no/ddc/projects/saab/asfouriyeh/annual-reports/index.html.

7 Omnia El Shakry (2015) and Pascale Ghazaleh (2019) provide an extensive overview of the debates on archives and writing histories in the Middle East today. I am especially inspired by the work of Mersal (2019), Mosallam (2014), Chatta (2020), Kaedbey (2014) and Altuğ (2021) among others.

8 A overview of the hospital’s annual reports between 1950 and 1960 does not show such a diagnosis.

9 Lebanon was one of the 24 countries from Africa, the Middle East and Asia surveyed in his study.
